# Heat Exposure and Maternal Health in the Face of Climate Change

**DOI:** 10.3390/ijerph14080853

**Published:** 2017-07-29

**Authors:** Leeann Kuehn, Sabrina McCormick

**Affiliations:** 1 Milken Institute School of Public Health, Department of Environmental and Occupational Health, George Washington University, Washington, DC 20052, USA; leeannkuehn@gmail.com; 2 School of Medicine and Health Sciences, Department of Physician Assistant Studies, George Washington University, Washington, DC 20052, USA

**Keywords:** climate change, maternal health, fetal health, heat exposure

## Abstract

Climate change will increasingly affect the health of vulnerable populations, including maternal and fetal health. This systematic review aims to identify recent literature that investigates increasing heat and extreme temperatures on pregnancy outcomes globally. We identify common research findings in order to create a comprehensive understanding of how immediate effects will be sustained in the next generation. Following the Preferred Reporting Items for Systematic Reviews and Meta-Analyses (PRISMA) guide, we systematically reviewed articles from PubMed and Cochrane Reviews. We included articles that identify climate change-related exposures and adverse health effects for pregnant women. There is evidence that temperature extremes adversely impact birth outcomes, including, but not limited to: changes in length of gestation, birth weight, stillbirth, and neonatal stress in unusually hot temperature exposures. The studies included in this review indicate that not only is there a need for further research on the ways that climate change, and heat in particular, may affect maternal health and neonatal outcomes, but that uniform standards for assessing the effects of heat on maternal fetal health also need to be established.

## 1. Introduction

Climate has direct effects on the health of diverse populations across the globe [1,2]. Changes in rainfall patterns and temperature patterns are resulting in far-reaching health effects [3] Vector and food-borne diseases, food insecurity, heatwaves and the effects of other extreme weather events make millions sick annually [4]. Heat is possibly the climate-related illness of greatest concern. In the United States, extreme heat events are causing more deaths than all weather-related fatalities combined [5]. High temperatures adversely impact the human body by interfering with its ability to dissipate heat and thermoregulate, leading to heat exhaustion and possibly heat stroke, a condition characterized by a core temperature ≥40.6 °C and central nervous system dysfunction [6]. 

Populations vulnerable to heat have most often been children under the age of five and adults over 65, individuals with pre-existing conditions, and those of low socioeconomic status who have limited ability to cope [7,8]. Urban populations are of central concern since the urban heat island effect is created when limited greenspace and larger proportions of impenetrable surfaces reflect heat in the surrounding environment, resulting in localized microclimates 4.4–5.6 °C above average temperatures [9,10]. However, there are other vulnerable populations who have not generally been of concern who deserve greater attention.

Despite research demonstrating the effects of heat on pregnant women, the effects of heat on them and their fetuses have often been left out of this discussion. The more recent assessment by the Intergovernmental Panel on Climate Change did explore the adverse effects of heat on maternal health outcomes [10]. The World Health Organization has named improving maternal and child health a continued focus in their Sustainable Development Goals, as targets from the Millennium Development Goals for maternal and child health were met. Despite improvements in reducing under age five child mortality and the global maternal mortality ratio, both efforts failed to meet the respective 66% and 75% reduction goals [11]. Up to 40.3% of deaths in children under five years of age occur in neonates [12], and preterm births account for 35% of neonatal mortality worldwide [1]. Preterm birth rates may reach 18% in some sub-Saharan African and South Asian countries, and only three countries globally successfully reduced preterm birth rates from 1990 to 2010 [1].

Pregnant women are susceptible to increasing ambient temperatures and heat waves since their ability to thermoregulate is compromised [13]. Furthermore, pregnancies are susceptible to complications at all stages of gestation. Such complications may affect maternal health, fetal health, perinatal health, or postnatal health of the mother and/or child [14], and are complex in both etiology and outcome. The sequelae of heat exposure on developing fetuses are not yet completely understood since the epidemiology behind many adverse fetal outcomes, including preterm delivery, is diverse [1]. Low birth weight was previously hypothesized as a consequence of sustained heat exposure and maternal heat stress [13]. While there is not conclusive evidence at this point, there may be a connection between adverse birth events and extreme deviation in ambient temperature. 

We conduct a systematic review to identify how extreme heat, specifically effects maternal, fetal, and neonatal health. There have been a handful of systematic reviews on the influences of climate change and variety of birth outcomes [14,15,16]. This review is an update and focused assessment of preterm labor, birth weight, and stillbirth as a result of ambient temperature exposures in a rapidly developing field of research. Additionally, this review specifically addresses the failure of existing heat health recommendations to capture heat-related morbidity in fetuses and pregnant women. This allows us to advance recommendations for pregnant women in the face of climate change by addressing insufficient public health recommendations for those populations during extreme heat events.

## 2. Materials and Methods 

In January, 2017, we conducted a systematic review of the literature, following the Populations of interest, Exposures, Comparators, and Outcomes (PECO) Statement model. We investigated impacts of climate change processes on maternal, fetal, and neonatal health by targeting publications in the PubMed database (6450 results) and Cochrane Review (0 results). As the original search in PubMed returned a comprehensive number of articles, no searches in additional database were deemed necessary. Our search included the following terms: human AND temperature (OR ambient temperature OR hot temperature OR heat wave OR climate change) AND pregnancy (OR pregnancy outcome OR birth weight OR preterm birth OR gestational age OR stillbirth). Papers were included on the basis of pertaining to maternal health, reproductive health, birth outcomes, child health, and climate change processes. We focused on maternal, fetal and child populations, with the given exposure of atypical climatic processes. The primary exposure and climate change topic of interest was heat and heat waves. The outcome of interest was a notable change in health outcome for any one of these populations. Papers were excluded if they did not examine human populations. Papers that focused on specific disease processes, such as the vertical transmission of viruses in pregnant women, the indoor ambient environment, or maternal and fetal outcomes unrelated to climate change or temperature were excluded. 

We examined each research article for the following criteria: population of exposure, nature of exposure, measured outcome, study type, location, and possible sources of bias. In exposure, we noted data on temperature measurements, duration of heat events, and severity of temperature exposure. For each type of outcome, we collected statistical outcomes, confidence intervals, and risk estimates, when provided. Confounding or mitigating factors, such as air pollution and socioeconomic variables, were also collected. Each article was examined for the possibility of bias, including selection bias, misclassification bias, detection bias, internal validity. Definitions for preterm birth, early birth, lower birth weight, and still-birth were used according to established literature. The World Health Organization defines preterm birth as occurring at less than 37 weeks of gestational age, and low birth weight as birth weight of less than 2500 g [17]. Early term birth is defined as 37–38 gestational weeks at birth. 

**Figure 1:**
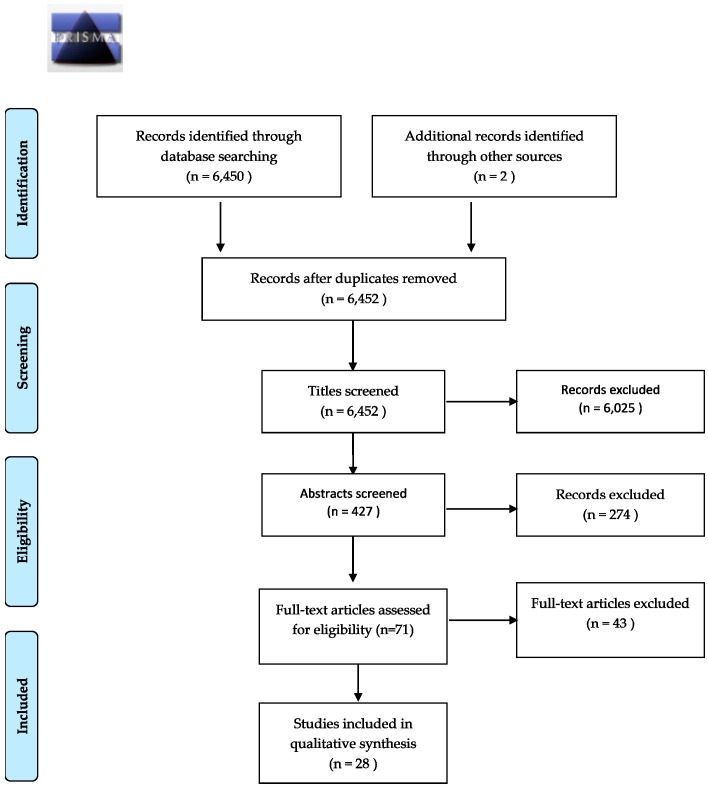
PRISMA flow diagram of study selection process.

The study selection proceeded according to Preferred Reporting Items for Systematic Reviews and Meta-Analyses (PRISMA) guidelines. Of 6450 initially screened studies, 28 met final selection criteria for this review. All search results were exported to RefWorks. The papers were screened initially by title, contents of abstract, and then by contents of full text by one reviewer. The search terms returned both reviews and original research articles; reviews were excluded from this literature review. As such, the final selection of articles is a combination of literature retrieved from the initial search terms, from reviewing reference lists of selected articles and linked citations from newly published literature, though only 1 source was returned via hand selection (see Figure 1).

Studies were selected based on their adherence to these search criteria, and were evaluated based on the strength of their findings and the risk of bias present in each study. We examined each research article for the following criteria: population of exposure, nature of exposure, measured outcome, study type, location, and possible sources of bias. In exposure, we reviewed method of temperature measurement, duration of heat events, and nature of temperature exposure. For each type of outcome, we collected statistical findings where provided. Inclusion of air pollution and socioeconomic variables were included in the final analysis of the study. In the event that a study was deemed to have high risk of bias, it would be removed from the literature review; however, no studies were deemed to have inappropriate risk of bias. 

Incidence or risk of preterm birth, early term birth or shortened gestation, birth weight, low birth weight, stillbirth, and neonatal heat-related distress all arose as relevant outcomes for temperature in this review. Furthermore, the intensity of the temperature exposure is wide-ranging. This review includes exposures measured over a variety of intervals, seasons, and extremes. Such variety in data collection necessitates a wide range of study designs.

## 3. Results

### 3.1. Birth Outcomes 

Table 1 shows study characteristics and whether heat exposure had a significant effect on the adverse outcomes examined within individual studies. Of the outcomes under investigation, preterm birth was the most common, investigated in 17 studies [18,19,20,21,22,23,24,25,26,27,28,29,30,31,32,33,34]. Preterm birth was defined as births occurring at less than 37 weeks of gestational age with a lower margin of 20 weeks, 22 weeks, or undefined in the studies. Heat was significantly correlated with increased risk or rate of preterm birth in 15 of 17 studies [19,20,21,22,23,24,25,26,27,28,29,30,31,32,34]; two studies found no significant effect [18,33]. A protective effect was demonstrated in Shenzhen, China for extreme heat exposure and preterm birth [30]. This result was in opposition to the other 13 studies and specifically to a study conducted in nearby Guangzhou [29]. Early term birth, or that occurring between 37 and 38 gestational weeks, was examined in a total of six studies [18,19,21,28,35,36]. In five of six studies, excess heat exposure was correlated with increased risk of early term birth [18,19,21,28,36]. 

Low birth weight at the time of delivery relative to gestational age was examined by five studies [33,34,37,38,39]. Of those studies, all five addressed heat outcomes [33,34,37,38,39]; three of five found significant correlations of increased heat exposure and low birth weight [34,37,38]. Seven studies examined the general effect of decreased birthweight as a result of ambient temperature exposure [31,34,35,38,40,41,42]. Six studies found significant negative correlations with birth weight at delivery [34,35,38,40,41,42].

Additionally, few studies address stillbirth rates. Only three studies evaluated the risk of stillbirth as a result of temperature exposure [19,31,43]. One study considered ambient temperatures and stillborn risk, but included two extreme temperature events in the analysis, one due to extreme heat [43]. The measures of stillbirth varied across each study. There were three main measurement definitions: a fetal death beyond 24 weeks of gestation, indicated by a lack of breathing after delivery [31], beyond 20 weeks of gestation [19], and gestation beyond 12 weeks [43]. Two of three studies found increasing rates of stillbirth with increasing ambient temperatures [19,43].

Lastly, two studies examining immediate neonatal impacts due to ambient temperature exposure were included in this review. Both demonstrated that heat waves increase stress in neonates, either as a function of heat-related neonatal intensive care unit (NICU) admissions [44], or through heat-related infant death [45]. Kakkad et al. [44] specifically explored NICU admissions on extreme heat days in Ahmedabad, India, where most births occur outside of hospitals and extreme heat is considered to be an excess of 42 °C; heat waves resulted in an increase of admissions. Basu et al. [45] uncovered effect modification within infant death rates during warm season months in California where Black children were more likely to die than other racial subgroups.

#### 3.1.1. Measuring Exposure

There was diversity in strategies to measure ambient temperature exposure. The intensity of the exposure variable was different, as the length of time captured by each exposure measurement varied across studies. Ambient temperature deviation was measured primarily as a departure from the historical mean, serving as the 50th percentile within the data. Deviations were considered on the order of 75th, 90th, 95th, and 99th percentiles for heat. The subdivisions varied among studies (see Dadvand et al. [36] and Schifano et al. [26]), which resulted differential categorization of moderate and extreme temperatures. 

Heat waves were defined differently within studies, whether by established definitions of heat events or as relative measures of measured data to historical temperature averages. Heat waves and extreme heat events were classified according to the length of time, by threshold temperature, or a combination therein (see Table 2). This review highlights the relative absence of a uniform definition of a heat wave within these studies. Notably, humidity and temperature measures such as apparent temperature and heat index (HI) were not universally analyzed within the studies. For example, Dadvand et al. [36] regarded HI to be a principal measure of exposure while Auger et al. [18] regarded humidity as a potential confounder. 

There was variable representation of exposure periods, either following a pregnancy for the full length of gestation (with appropriate measures to avoid a fixed cohort bias, as discussed in Strand et al. [46] for examining temperature effects within a short time frame. Specific time frames range from the week preceding birth, three weeks, one month, or throughout the course of the warm season with births occurring within that three-month interval. These sub-full gestation studies all investigated preterm birth or shortened gestation. Heat findings were significantly correlated within all sub-full gestation exposure periods, with the exception of preterm birth within a one-week exposure in Auger et al. [18], counter to the findings of Basu et al. [23]. 

Four studies [38,41,42,43] measured long-term changes in mean annual temperature, and as such compiled years averages temperature. The exposure assessments conducted in these studies address the long-term consequences of changing climate, rather than specific extreme heat events, though one did include analysis of an extreme heat event and cold event [43].

#### 3.1.2. Exposure/Outcome Relationship

Overall, heat exposure was highly correlated with adverse outcomes, 32 significant findings in 36 investigations of adverse outcomes within 28 studies. In the instance that both temperature measures were significantly associated with outcomes, the hazards distribution followed a nonlinear U shape (see Table 1). These findings primarily occurred in studies examining preterm birth or early term birth, and occurred in exposure periods ranging from the full length of gestation to exposures in the last week of gestation. Heat was largely correlated with a decrease in birth weight, decreased gestational length, increased risk of still birth, and increases in neonatal stress and mortality. While stillbirths followed a linear relationship, birthweight and gestational length and temperature were nonlinear. 

### 3.2. Critical Exposure Window

The literature has not established a critical period of maternal sensitivity to hot temperatures [26,27,30,31,34,38]. At the time of this review, no preterm birth or birth weight susceptibility window period has been established. Some studies in this review measure temperature exposure over the course of gestation while others examine temperatures in the short term, such as a week or month leading to delivery. Due to the variability in measured exposure windows, there is no consensus in critical window periods. Of studies that measure the full length of gestation, each trimester was separately identified as the primary window. 

There is limited evidence for critical heat-related window periods. Heat exposures may have immediate effects on birth outcomes, as well as delayed (lagged) or cumulative effects on birth outcomes. When specifically examining the last week of pregnancies with preterm deliveries, Basu et al. [23] revealed the most significant increase of preterm risk six days prior to delivery, while exposure on the day prior to delivery also poses elevated risk; Kent et al. [24]. Exposure lag periods of up to several weeks were found to be significantly correlated with adverse outcomes. Vicedo-Cabrera et al. [27] found a four-week lag in preterm births following extreme heat exposure. Dadvand et al. [36] found decreased gestational age at birth to be associated with a lag interval of 5 days after heat exposure. The cumulative effect of temperature exposure must also be considered when addressing exposure intervals, such as in exposures occurring over consecutive days [18,20,26] and increase in risk at moderate temperatures following an extreme exposure [22].

### 3.3. Duration versus Threshold

There has been some question regarding which drives adverse outcomes more, the acute level of exposure or the duration of exposure. In this review, we see that surpassing a temperature threshold during a heat event may be the most critical component to adverse birth outcomes during an extreme heat event as noted in Wang et al. [20]. While the duration of a heat event was examined as a function of preterm birth hazard, the data show that surpassing historical temperature percentiles in each city resulted in an adverse birth outcome of some kind across all studies. The length of exposure varied from a one-day impact to cumulative exposures of consecutive days or days spread over the course of a month. Even with heterogeneity in exposure lengths, heat waves and increased ambient heat load were correlated with the prevalence or risk of adverse birth outcomes. Initial adverse birth outcomes may be triggered at a critical temperature threshold and subsequent cumulative heat exposure brought on by the duration of the event are likely associated with increasing morbidity in susceptible groups.

The risk for adverse outcomes at a specific temperature will not be uniform in other climates, as mean temperature and humidity may vary dramatically between climates. The women in these studies were adapted to the temperatures typical of their respective climates, and heat waves represented significant deviations from their climate norms. Heat morbidity thresholds are less extensively studied than mortality thresholds and may vary regionally [28]. The issue of acclimatization is further exaggerated by the timing of heat exposures, wherein heat waves earlier in the warm season may trigger adverse outcomes more readily than at the end of season after acclimatization has taken place [23]. Regardless of the relative warmth of the climate, excessive heat exposure resulted in adverse birth outcomes across the majority of studies in this review.

## 4. Discussion

This systematic review demonstrates that extreme heat exposure affects fetal outcomes, ranging from stillbirth rates to birth weight and gestational age. This trend is present in research regarding reduced gestational age and preterm birth. The specifics of this review represent some clear conclusions regarding actions that should be taken to prevent adverse effects of heat on pregnant women. 

First, public health agencies should specify that their warnings apply to pregnant women. This is complicated by the lack of uniformity in defining a heat wave. The definition of a heatwave alters the amount of heat-related morbidity captured within an exposure period. Recent meta-analysis demonstrates that correlations between mortality and specific heat wave definitions vary by the temperature threshold and the duration of the event [47]. The most important metric for determining both mortality and morbidity from heatwaves is intensity [20,48]. The inclusion of humidity and other meteorological factors may not increase prediction of heat-related morbidities [24] or may cause a direction of effect that was counterintuitive [49]. The most important component of generating an appropriate definition of a heatwave is consideration of average local temperatures [24,47,48,49,50] but in correlation with the specific health profile of the target population, focusing on the health risks posed by excess heat exposure [51]. 

When attempting to reduce heat-related illness through public health warnings, the timing of the heat wave should be taken into consideration. Heat events occurring earlier in the warm season cause an increase in adverse outcomes relative to comparable events occurring later. As such, the relative acclimatization of the population should be considered as an additional factor when generating heat warnings. Furthermore, these results may not appear initially, but rather as a lagged effect, with adverse outcomes appearing from immediately after exposure to as late as one month post exposure [23,27]. While some extreme heat exposures resulted in acute outcomes, exposures to moderate temperature spikes may results in an adverse birth outcome delayed by a longer time interval. 

Second, urban planning may also become a necessary tool to prevent adverse birth outcomes. Urban heat is affected by albedo (reflectivity) and land use patterns. More specifically, as land use increases and reflectivity increases, the relative magnitude of UHI will also increase [52]. Increasing greenspace may reduce the ambient temperature of a given area, particularly through increasing canopy cover [52]. Without adequate urban planning and adaptation, cities will rely on air conditioning, an energy-intensive mitigation strategy, to reduce exposure [53]. 

Yet, air pollution has been correlated with all-cause mortality [54] and low birth weight [55]. Levels of fine particulate matter (such as PM2.5) may increase in low and middle income countries (LMICs) as well as developing countries, serving to further exacerbate adverse fetal health risks [55]. Increasing air pollution levels appear to cause increased maternal stress [56] that is exacerbated in heat waves when pollutant concentrations rise [3], further impacting fetal stress. While most studies accounted for air pollution as a potential confounder, air pollution coupled with heat may directly impact both maternal health and birth outcomes [37]. Therefore, strategies to address heat exposure for pregnant women should also reduce both the impact of extreme heat and air pollution through urban design and implementation of heat warning systems [52,53]. Greenspace should be expanded within urban areas, not only as it reduces the impacts of UHI, but since proximity to greenspace has additionally been linked to lower PM2.5 exposures in pregnant women [57], and may reduce risk for adverse birth outcomes. 

Finally, identifying the most effective strategies to prevent adverse outcomes would ideally rely on an improved understanding of the primary causes underlying adverse birth outcomes from heat. However, they are currently not well understood, as the etiologies of low birth weight and preterm birth are complex. There are a number of theories regarding this connection, such as increased maternal heat production [13], increased weight gain, and the burden of the fetus overwhelming a pregnant woman's ability to tolerate heat stress, resulting in low birth weight. Increasing heat stress may cause uterine constriction, and dehydration may result in decreased uterine blood flow [58]. Inflammation may also contribute to low birth weight or preterm birth [58]. Díaz et al. [39] argue that preterm birth and low birth weight outcomes must be considered separately due to the high correlation between preterm birth and low birthweight. Socioeconomic factors may further play a role, as psychological stress, social stress, and housing instability may further increase risk for preterm birth [58,59].

While there has been significant progress on heat and birth research in the last five years, a number of limitations remain. We only included articles that are written in English. While this obviously offers an imbalanced representation of the literature, we do not believe it is a biased review since many studies in non-English speaking countries are still represented. Several studies report effect modification by socioeconomic factors, such as race, income, and profession [26,35]; these factors are not included in analysis universally in the data sets. Furthermore, there is a widespread lack of information regarding home air conditioning access, or other resources that serve as mitigating factors in heat exposure [35]. These factors may serve as confounders within studies, bias findings towards the null, or cause discord in results among studies. The protective effect of extreme heat (99th percentile) on preterm birth observed by Liang et al. [30] demonstrates that socioeconomic data and air conditioner use may impact preterm birth in Shenzhen, China differently from other cities in ways not yet quantified. However, this review of the literature is not a meta-analysis, and therefore cannot draw further statistical significance of heat impacts on birth outcomes beyond the findings of the original studies. 

## 5. Conclusions

The World Health Organization and the World Meteorological Association have suggested that the heat exposure should be taken into consideration in both clinical care and public health programming. Vulnerability will play an additional role in heat-stress susceptibility. Current literature largely refers to heat-susceptible individuals as poor, elderly, young children, minority groups, outdoor workers, individuals with chronic respiratory or cardiovascular disorders, socially isolated individuals, and living in an urban heat island [2,7,60,61]; notably, pregnant women are not included. However, limited thermoregulatory abilities warrants, dependent on medical care access, the inclusion of pregnant women as a vulnerable class in the face of heat exposure [13,38]. Therefore, when considering the exaggerated impacts of heat, pregnant women must also be included as an at-risk class. Vulnerability and warnings should be specified to local context. Heat tolerance and acclimatization may affect the heath of pregnant women and fetuses. The threshold temperature that results in significant effects varies by climate since women in each region have acclimatized to a relative threshold for extreme heat. 

Due to climate change, increasing heat waves are likely unavoidable in the coming century. Heat exposure promises to affect population health, such as developing fetuses and pregnant women. We are likely to see an increase in preterm birth, a decrease in birth weight, and an increase in stillbirth rates. After birth, neonates may be susceptible to heat-related morbidity and mortality, though additional research is required. These findings indicate the importance of ongoing public health efforts to combat local effects of climate change.

## Figures and Tables

**Table 1:** Study characteristics, significance within individual study findings, and exposure windows.

Author	Location	Sample Size	Early Birth	Preterm Birth	Low Birth Weight	Reduced Birth Weight	Stillbirth	Postnatal Outcomes	Exposure Window
Auger et al. 2014 [18]	Montreal, Canada	206,929	Yes	No	-	-	-	-	1 week prior to delivery
Strand et al. 2012 [19]	Brisbane, Australia	101,870	Yes	Yes	-	-	Yes	-	4 weeks prior to delivery
Wang et al. 2013 [20]	Brisbane, Australia	50,848	-	Yes	-	-	-	-	Up to 3 months prior to delivery
Schifano et al. 2016 [21]	Rome, Italy and Barcelona, Spain	78,633 (Rome), 27,255 (Barcelona)	Yes	Yes	-	-	-	-	Full gestation
Vicedo-Cabrera et al. 2014 [22]	Valencia, Spain	20,148	-	Yes	-	-	-	-	3 weeks prior to delivery
Basu et al. 2010 [23]	California, USA	60,000	-	Yes	-	-	-	-	1 week prior to delivery
Kent et al. 2014 [24]	Alabama, USA	60,466	-	Yes	-	-	-	-	1 week pior to delivery
Arroyo et al. 2016 [25]	Madrid, Spain	298,705	-	Yes	-	-	-	-	Full gestation
Schifano et al. 2013 [26]	Rome, Italy and Barcelona, Spain	132,691	-	Yes	-	-	-	-	Full gestation
Vicedo-Cabrera et al. 2015 [27]	Stockholm, Sweden	95,069	-	Yes	-	-	-	-	4 weeks prior to delivery
Cox et al. 2016 [28]	Flanders, Belgium	807,835	Yes	Yes	-	-	-	-	Full gestation
He et al. 2016 [29]	Guangzhou, China	838,146	-	Yes	-	-		-	Full gestation
Liang et al. 2016 [30]	Shenzhen, China	1,040,638	-	Yes	-	-	-	-	Full gestation
Bruckner et al. 2014 [31]	Uppsala, Sweden	13,657	-	Yes	-	No	No	-	Full gestation
Mathew et al. 2017 [32]	Alice Springs, Australia	16,870	-	Yes	-	-	-	-	3 weeks prior to delivery
Wolf and Armstrong 2012 [33]	Brandenburg and Saxony, Germany	291,517	-	No	No	-	-	-	Full gestation
Kloog et al. 2015 [34]	Massachusetts, USA	450,407	-	Yes	Yes	Yes	-	-	Full gestation
Ngo and Horton 2016 [35]	New York, USA	514,104	No	-	-	Yes	-	-	Full gestation
Dadvand et al. 2011 [36]	Barcelona, Spain	7585	Yes	-	-	-	-	-	1 week prior to delivery
Dadvand et al. 2014 [37]	Barcelona, Spain	6438	-	-	Yes	-	-	-	Full gestation
Molina and Saldarriaga 2017 [38]	Bolivia, Colombia, and Peru	86,000	-	-	Yes	Yes	-	-	Full gestation
Díaz et al. 2016 [39]	Madrid, Spain	298,705	-	-	No	-	-	-	Full gestation
Poeran et al. 2016 [40]	Netherlands	1,460,401	-	-	-	Yes	-	-	Full gestation
Jensen and Sørensen 2013 [41]	Global Data: 125 populations	Not Reported	-	-	-	Yes		-	Full gestation
Wells and Cole 2002 [42]	Global Data: 140 populations	Average: 97,237	-	-	-	Yes	-	-	Full gestation
Fukuda et al. 2014 [43]	Japan	Not Reported	-	-	-	-	Yes	-	Full gestation
Kakkad et al. 2014 [44]	Ahmedabad, India	2025	-	-	-	-	-	Yes	After delivery
Basu et al. 2015 [45]	California, USA	12,356	-	-	-	-	-	Yes	After delivery

**Table 2:** Select heat wave and extreme heat definitions from reviewed articles.

**Heat Wave Definitions Tested in Reviewed Articles**		
Kakkad et al. 2014 [44]	1. When normal temperature is <40 °C:	Heat wave if 5–6 °C increase
		Severe heat wave if 7 °C+ increase
	2. When normal temperature is ≥40 °C:	Heat wave if 4–5 °C increase
		Severe heat wave if 6 °C+ increase
	3. If temperatures exceed 45 °C:	Severe heat wave
Wang et al. 2013 [20]	1. Exceed 90th temperature percentile (30.38 °C) over a 2, 3, or 4 day minimum duration	
	2. Exceed 95th temperature percentile (31.32 °C) over a 2, 3, or 4 day minimum duration	
	3. Exceed 99th temperature percentile (32.52 °C) over a 2, 3, or 4 day minimum duration	
Mathew et al. 2017 [32]	Temperature >40 °C for 3 consecutive days	
**Extreme Heat Definitions Measured in Reviewed Articles:**		
Auger et al. 2014 [18]	1. ≥32 °C for 3 consecutive days	
	2. ≥32 °C for 1, 2, 3, and 4 to 7 days	
Dadvand et al. 2011 [36]	Heat-humidity index >90th, >95th, or >99th percentile for 1 day	
